# Sub-coronary ROSS operation in a young man with quadricuspid aortic valve and anomalous aortic origin of the left coronary artery: A case report

**DOI:** 10.1097/MD.0000000000041302

**Published:** 2025-01-17

**Authors:** Li Xu, Yueqiu Su, Zhou Leng

**Affiliations:** a Operating Room, Department of Anesthesiology, West China Hospital, Sichuan University/West China School of Nursing, Sichuan University, Chengdu, Sichuan, China; b Department of Anesthesiology, West China Hospital, Sichuan University/West China School of Nursing, Chengdu, Sichuan, China; c Department of Anesthesiology, West China Hospital, Sichuan University, Chengdu, Sichuan, China.

**Keywords:** quadricuspid aortic valve, ROSS procedure, unroofing procedure

## Abstract

**Rationale:**

Quadricuspid aortic valve (QAV) is a rare condition with a very low incidence. Anomalous aortic origin of a coronary artery (AAOCA) is the most prevalent form of coronary anomaly. One variant of AAOCA is the anomalous aortic origin of the left coronary artery from the right coronary sinus (L-AAOCA). It is even rarer for QAV to occur in combination with AAOCA.

**Patient concerns:**

In this case report, we present a patient who was diagnosed with QAV and L-AAOCA using preoperative transesophageal echocardiography and 3-dimensional computed tomography (3D-CT).

**Diagnoses:**

Cardiac-enhanced CT showed an anomalous aortic origin of the left coronary artery from the right coronary sinus with an intramural course.

**Interventions:**

The patient underwent sub-coronary ROSS procedure along with the unroofing procedure.

**Outcomes:**

The timely diagnosis of this incorporated cardiac anomaly, or rather its accidental discovery, led to successful surgical correction, eliminating the future risk of cardiac ischemia or SCD.

**Lessons:**

The timely diagnosis of this incorporated cardiac anomaly, or rather its accidental discovery, led to successful surgical correction, eliminating the future risk of cardiac ischemia or SCD.

## 1. Introduction

Quadricuspid aortic valve (QAV) is a rare condition, with an estimated incidence of 0.01% to 0.04%.^[1]^ It is characterized by the aortic valve having 4 cusps of varying sizes, instead of the more commonly observed 3 symmetrical cusps. QAV is not typically diagnosed at birth and is nonpathological, but it can develop into aortic stenosis and/or regurgitation over time. Approximately 32% of patients with QAV also have an additional congenital heart defect, with anomalous aortic origin of a coronary artery (AAOCA) being the most common at 10%. AAOCA includes a range of variants, each of which is seen in <1% of the general population. According to reports, patients with abnormal aortic valves have an almost double prevalence of AAOCA compared to patients with tricuspid aortic valves.^[2]^ The most prevalent type of AAOCA is the right coronary artery from the left sinus (R-AAOCA), with a prevalence of 0.33%, compared to the lower prevalence of 0.12% of L-AAOCA. Hence, in the current body of work, the combination of QAV and R-AAOCA has been documented more frequently. Patients with L-AAOCA generally have a higher risk of myocardial ischemia compared to those with R-AAOCA, as the left coronary artery (LCA) originating from the right sinus is more likely to have an interarterial and intramural course.^[3]^ Moreover, there may be a correlation between the QAV and aortic dilation and aneurysms,^[4]^ which further worsens the impact of QAV with L-AAOCA. This case report details a successful sub-coronary ROSS and unroof procedure used to treat a patient with QAV and L-AAOCA with intramural course.

## 2. Case presentation

**Figure 1.**
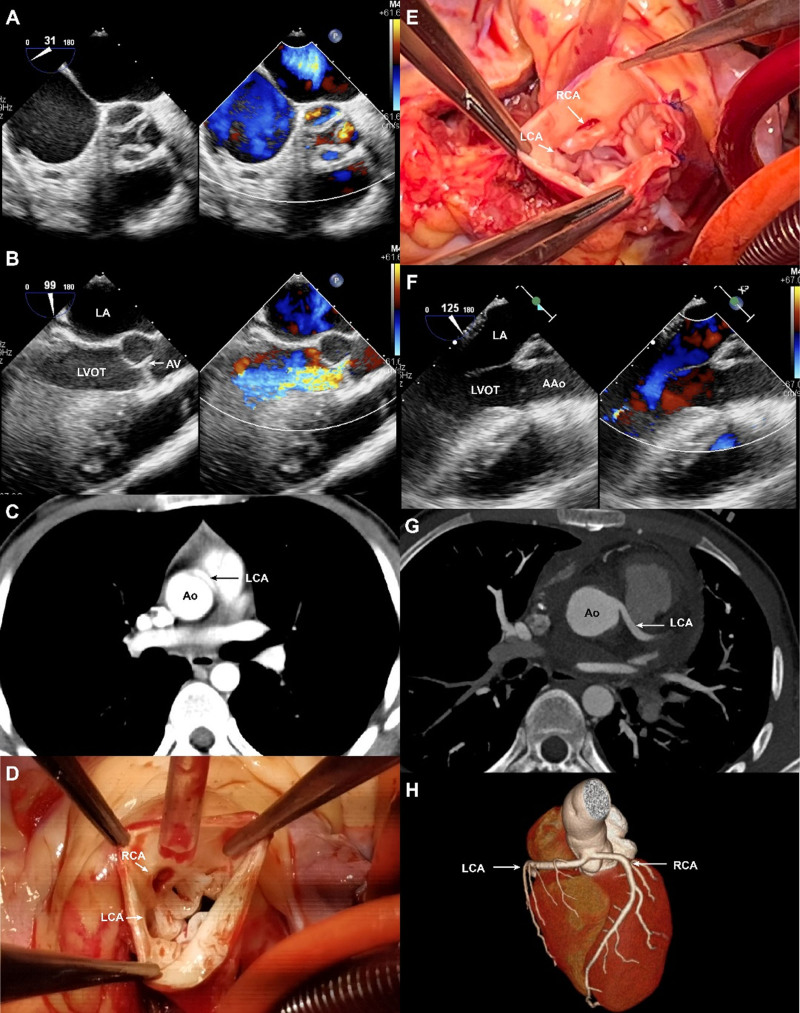
(A) A QAV in the short-axis view of the aortic root, (B) Aortic annular diameter was measured to be approximately 22 mm, (C) Cardiac-enhanced CT showed an anomalous aortic origin of the left coronary artery from the right coronary sinus with an intramural course, (D) The presence of QAV and double coronary artery ostia in the right sinus of Valsalva, (E) Pulmonary artery wall affixed below the coronary artery, (F) smooth LVOT, (G) and (H) Confluent ostium of coronary arteries and normal course of the coronary arteries. CT = computed tomography, LVOT = left ventricle outflow track, QAV = quadricuspid aortic valve.

Our hospital received a 22-year-old male patient who had been found to have a cardiac murmur during a routine physical examination, despite not showing any symptoms. Figure 1A showed a QAV in the short-axis view of the aortic root, while severe aortic stenosis (AS) and severe aortic regurgitation (AR) were revealed in the long-axis view of the left ventricle outflow track (LVOT). The aortic annular diameter was measured to be approximately 22 mm by transesophageal echocardiography in Figure 1B. Cardiac-enhanced CT showed an anomalous aortic origin of the left coronary artery from the right coronary sinus with an intramural course (Fig. 1C).

After thorough discussions, the decision was made to proceed with the sub-coronary ROSS procedure along with the unroofing procedure. Once the routine cardiopulmonary bypass was established, a transverse aortic incision was made superior to coronary arteries, which revealed the presence of QAV and double coronary artery ostia in the right sinus of Valsalva (Fig. 1D). The aortic cusps were removed, and the intramural segment of LCA was unroofed. The aortic annulus was cut lengthwise along the noncoronary sinus, and a patch of the patient’s own pericardium was used to expand the aortic annulus. The autologous pulmonary valve was connected to the distal LVOT at the aortic valve annular, while the pulmonary artery wall was affixed below the coronary artery (Fig. 1E). During the intraoperative transesophageal echocardiography, it was observed that the LVOT was smooth and there was no aortic regurgitation (Fig. 1F). After the surgery, a postoperative 3D-CT revealed a confluent ostium of coronary arteries and normal course of the coronary arteries (Fig. 1G, H).

## 3. Discussion

QAV is usually found as a standalone abnormality, and when adults are affected, they often show signs of aortic valve dysfunction, such as regurgitation, in their fifties and sixties. Hurwitz and Roberts described 8 anatomic variations of QAV.^[5]^ Type A consists of 4 equal-sized cusps. Type B consists of 3 equal-sized cusps and 1 smaller cusp. Type C consists of 2 equal large and 2 equal smaller cusps. Type D consists of 1 large cusp, 2 mid-sized cusps and 1 smaller cusp. Type E consists of 3 equal-sized cusps and 1 large cusp. Type F consists of 2 equal larger cusps and 2 unequal smaller cusps. Type G consists of 4 unequal cusps, Type H consists of 1 large cusp, 1 mid-sized cusp and 2 equal-sized smaller cusps. Type B is the most common variation among the 8 variations, and it is consistent with the anatomy of our patient. It has been proposed that the Type B has a higher likelihood of developing valve dysfunction due to the single undersized cusp progressively leading to uneven stress distribution, trauma, and mal-coaptation,^[6]^ this may be the reason for our patient need surgical intervention at the age of 22 years.

AAOCA is the second most common cause of sudden cardiac death (SCD) in otherwise healthy individuals, with R-AAOCA occurring 4 to 5 times more frequently than L-AAOCA. However, L-AAOCA has been more strongly linked to sudden death and ischemia. AAOCA can be treated with a large spectrum of interventions, but for L-AAOCA with high-risk anatomical features like LCA interarterial and intramural course, surgical intervention is strongly recommended.^[2]^

The concurrent presence of AAOCA and QAV, 2 pathological states, is an uncommon event that deserves deeper exploration to elucidate their interplay and any possible correlation. Given the scarcity of documented cases in scholarly sources, it remains uncertain whether there is a definitive connection between these conditions or if they occur separately.^[7]^

Hereditary aortic valve disorders typically stem from anomalies in the truncal separation process, as the heart tube folds to create dual pumping mechanisms and great arteries, a stage that typically occurs between the 4th and 8th week of embryonic development. The rightward migration of the atrioventricular canal and the leftward shift of the ventricular septum align each ventricle with the systemic circulation. The formation of the distal arteries precedes this alignment. These inherited valve conditions often manifest as bicuspid valves, which are found in about 2% of the population. Less commonly, monocuspid or quadricuspid valves have been identified both in clinical settings and in postmortem examinations, with a frequency of <0.02%. However, it’s worth noting that the presence of additional aortic cusps can exceed 4.^[8]^ Approximately 32% of QAV patients have an additional congenital heart defect, and AAOCA is the most common, accounting for 10%. Due to the higher prevalence of R-AAOCA compared to L-AAOCA, the combination of QAV with L-AAOCA is rarely mentioned in the literature. The presentation of our patient with a QAV and L-AAOCA with LCA interarterial and intramural course is uncommon, as this patient does not exhibit any symptoms related to AS and AR or coronary occlusion.

## Author contributions

**Conceptualization:** Li Xu, Yueqiu Su, Zhou Leng.

**Data curation:** Li Xu, Yueqiu Su, Zhou Leng.

**Formal analysis:** Yueqiu Su, Zhou Leng.

**Investigation:** Li Xu, Yueqiu Su, Zhou Leng.

**Methodology:** Li Xu, Yueqiu Su, Zhou Leng.

**Project administration:** Li Xu, Yueqiu Su, Zhou Leng.

**Resources:** Li Xu, Yueqiu Su, Zhou Leng.

**Software:** Yueqiu Su.

**Supervision:** Li Xu, Yueqiu Su, Zhou Leng.

**Validation:** Li Xu, Yueqiu Su, Zhou Leng.

**Visualization:** Li Xu, Yueqiu Su, Zhou Leng.

**Writing – original draft:** Li Xu, Yueqiu Su, Zhou Leng.

**Writing – review & editing:** Li Xu, Yueqiu Su, Zhou Leng.
